# Association between serum 25-hydroxyvitamin D levels and pulmonary function in elderly patients with chronic kidney disease: correlation analysis and exploratory predictive modelling

**DOI:** 10.3389/fendo.2026.1777106

**Published:** 2026-03-24

**Authors:** Xuan Dai, Min Zhou, Qi Gao, Lianfang Yuan, Xiuwen Jiang, Jiarui Li, Li Zhang, Chan Guo, Naigang Gu

**Affiliations:** 1 Tianjin NanKai Hospital, Tianjin Medical University, Tianjin Key Laboratory of Acute Abdomen Disease Associated Organ Injury and ITCWM Repair, Institute of Integrative Medicine for Acute Abdominal Diseases, Tianjin, China; 2 Tianjin Medical University School of Nursing, Tianjin, China; 3 NHC Key Lab of Hormones and Development and Tianjin Key Lab of Metabolic Diseases, Tianjin Medical University Chu Hsien-I Memorial Hospital & Institute of Endocrinology, Tianjin, China; 4 Tianjin NanKai Hospital, Tianjin Medical University, Tianjin, China

**Keywords:** 25-hydroxyvitamin D [25(OH)D], chronic kidney disease, DLCO%, FEV1%, pulmonary function

## Abstract

**Background:**

Vitamin D has emerged as a significant indicator influencing pulmonary function, yet the complexity of how vitamin D affects the pulmonary function among patients with chronic kidney disease (CKD) remains incompletely elucidated. To preliminarily explore this issue, we observed the relationship between serum 25-hydroxyvitamin D [25(OH)D] levels and pulmonary function indicators in CKD patients, and initially explored the predictive model for pulmonary function abnormalities.

**Methods:**

This single-center cross-sectional study enrolled 90 CKD patients hospitalized in the Department of Nephrology, Tianjin Nankai Hospital, from July 2025 to November 2025. Serum 25(OH)D levels were measured, and pulmonary function testing was performed. Main indices included forced expiratory volume in 1 second (FEV1), percent predicted FEV1 (FEV1%), FEV1/FVC, and small-airway indices (FEF25/50/75). Diffusing capacity for carbon monoxide percent predicted (DLCO%) and diffusion impairment grade were also evaluated. Spearman correlation was used to assess associations between 25(OH)D and the above indices; multivariable linear regression was used to adjust for potential confounders. Random-forest classification models were further developed for exploratory prediction of pulmonary function abnormalities and to evaluate model performance.

**Results:**

Serum 25(OH)D levels were positively correlated with multiple pulmonary function indices, particularly predicted FEV1% (r = 0.463, P < 0.001) and DLCO% (r = 0.611, P < 0.001). Serum 25(OH)D was negatively correlated with diffusion impairment grade (r = -0.585, P < 0.001), indicating that higher 25(OH)D levels were associated with less severe diffusion impairment. In random-forest analyses, both models for predicting ventilatory impairment and diffusion impairment demonstrated certain specificity; sensitivity was higher for the FEV1% model, but overall specificity was limited, suggesting room for improvement when predicting pulmonary function abnormalities based solely on variables included in this study.

**Conclusions:**

Vitamin D deficiency was common in CKD patients, and serum 25(OH)D levels were independently associated with multiple pulmonary function indices, especially diffusion-related indices. As a cross-sectional study, causality cannot be inferred. Whether vitamin D supplementation can improve pulmonary outcomes requires further validation in prospective cohorts and randomized controlled trials.

## 1 Introduction

Chronic kidney disease (CKD) is a global health problem, with an adult prevalence as high as 10%-15% (1). CKD not only damages renal function (2), but also affects pulmonary function through multiple pathophysiological pathways (3) The mechanisms are complex and involve a micro-inflammatory state and oxidative stress induced by accumulation of uremic toxins, pulmonary interstitial edema and pleural effusion due to volume overload, electrolyte and acid-base disturbances, and respiratory muscle weakness caused by renal anemia and malnutrition (4, 5). These factors collectively result in predominantly restrictive ventilatory dysfunction and impaired diffusion capacity. (6). This places CKD patients at higher risk of pulmonary dysfunction, especially in advanced CKD, where declining pulmonary function markedly increases complications and is associated with adverse outcomes (7).

25(OH)D deficiency is relatively common in CKD and may worsen with declining kidney function (8). As estimated glomerular filtration rate (eGFR) decreases, renal 1-α-hydroxylase activity is significantly reduced, hindering conversion of vitamin D to its active form 1,25(OH)_2_D_3_ (9). In addition, urinary protein loss, insufficient sunlight exposure, and elevated fibroblast growth factor 23 (FGF23) levels together cause 25(OH)D deficiency as high as 70%-90% among CKD stage 3–5 patients. 25(OH)D receptors are widely distributed in alveolar epithelium and airway smooth muscle, and 25(OH)D exerts multiple lung-protective effects, including inhibition of tumor necrosis factor-α (TNF-α) and interleukin-6 (IL-6), promotion of surfactant synthesis, and regulation of the renin-angiotensin system (RAS) (10). These observations suggest a potential link between vitamin D and pulmonary function changes.

At present, evidence on the association between 25(OH)D levels and pulmonary function in CKD (including ventilatory function, small-airway indices, and diffusion function) remains limited, and existing studies differ in study populations, selected indices, and control of confounders. Therefore, it is necessary to further and systematically evaluate associations between 25(OH)D and multidimensional pulmonary function indices in CKD patients and to explore potential clinical implications.

Based on data from hospitalized CKD patients, this study used correlation analysis and multivariable regression to evaluate the independent associations between 25(OH)D and pulmonary function indices, and further constructed random-forest models for exploratory prediction of pulmonary function abnormalities.

## 2 Materials and methods

### 2.1 Study population and sample source

This single-center cross-sectional study included CKD patients hospitalized in the Department of Nephrology, Tianjin Nankai Hospital, from July 2025 to November 2025. Sample size estimation was based on a pilot study and relevant literature (8) With a moderate effect size (r ≥ 0.3), α = 0.05, and β = 0.2, the minimum required sample size was 84. Considering a 20% potential attrition/incomplete-data rate, the planned sample size was 105. A total of 90 eligible CKD patients were ultimately included, meeting basic requirements for regression analyses. CKD staging followed the KDIGO 2012 guideline based on eGFR: stage 1, eGFR ≥ 90 with markers of kidney damage; stage 2, eGFR 60–89 with markers of kidney damage; stage 3, eGFR 30-59; stage 4, eGFR 15-29; stage 5, eGFR < 15 or maintenance dialysis.

Inclusion criteria:

1. Age ≥ 18 years.
2. Meeting KDIGO diagnostic criteria for CKD: any of the following persisting for >3 months: (1) eGFR < 60 mL/min/1.73 m², or eGFR ≥ 60 with clear markers of kidney damage (e.g., proteinuria for ≥3 months); (2) imaging evidence of small kidneys or reduced cortical thickness; (3) renal pathology showing fibrosis or atrophy.
3. Provision of written informed consent.

Exclusion criteria:

1. Age < 18 years.
2. Expected survival < 6 months.
3. Severe pulmonary infection or history of surgery within the past 3 months.
4. Known active pulmonary disease (e.g., acute exacerbation of COPD, uncontrolled asthma, active pulmonary tuberculosis, lung cancer, acute phase of interstitial lung disease, severe pulmonary arterial hypertension, recent pulmonary embolism).
5. Use within the past 1 month of systemic glucocorticoids or other medications that significantly affect immune/inflammatory status (e.g., biologics, immunosuppressants), unless used for primary CKD etiologies such as lupus nephritis with a stable dose for >3 months.
6. Severe liver disease affecting vitamin D metabolism/function (e.g., cirrhosis Child-Pugh class B/C), severe hyperthyroidism, malabsorption syndrome, or sarcoidosis.
7. Acute heart failure or myocardial infarction, stroke, or shock within the past 3 months.
8. Recent use of vitamin D or active vitamin D preparations.
9. Inability to cooperate with pulmonary function testing.
10. Pregnancy or lactation.
11. Uncontrolled hypertension (SBP ≥ 200 mmHg and/or DBP ≥ 100 mmHg).

The study was approved by the hospital medical ethics committee (IRB No.: NKYY_YXKT_IRB_2025_091_01) and was conducted in strict accordance with the principles of the Declaration of Helsinki.

### 2.2 Data collection

Baseline data included age, sex, height, weight, BMI, smoking history, CKD stage, and comorbidities (hypertension, diabetes, coronary heart disease, heart failure, and autoimmune disease).

Laboratory indices: Fasting venous blood was collected in the early morning. Serum 25(OH)D levels were measured by chemiluminescent immunoassay (CLIA). Serum creatinine (Crea), eGFR, intact parathyroid hormone (iPTH), calcium (Ca), phosphate (P), hemoglobin (Hgb), albumin (ALB), C-reactive protein (CRP), and the urinary albumin-to-creatinine ratio (ACR), among other indicators, were measured concurrently.

Pulmonary function measurement: Data were collected using a Jaeger pulmonary function testing system (Germany). Environment and preparation: room temperature was maintained at 22-25 °C; large meals were prohibited within 2 hours prior to testing; intake of alcohol, cola, coffee, tea, etc. was prohibited; smoking was prohibited within 1 hour prior to testing; and strenuous exercise was avoided within 30 minutes prior to testing. Testing was conducted by professionally trained technicians. During testing, subjects sat upright without leaning on the backrest, with feet flat on the floor and the head in a natural horizontal position or slightly extended. Main indices included FVC, FEV1, FEV1% predicted, FEV1/FVC, small-airway indices (FEF25/50/75), and hemoglobin-corrected DLCO% predicted. Diffusion impairment was graded based on DLCO% predicted: normal ≥ 80%, mild 60%-79%, moderate 40%-59%, and severe < 40%. Quality control: after resting for 15 minutes, tests were performed in a seated position with a nose clip. Three acceptable curves meeting ATS/ERS grade-A quality criteria were obtained, and the curve with the best FEV1 was used for analysis. If none of the three tests met criteria, additional tests were performed, typically not exceeding eight attempts. The best value of each index was recorded for analysis to ensure data stability and reproducibility.

### 2.3 Statistical analysis

Analyses were performed using SPSS version 26.0. Normally distributed continuous variables are presented as mean ± standard deviation, and non-normally distributed variables as median (interquartile range). Categorical variables are presented as number (%). Diffusion impairment grade was coded as 0 (normal), 1 (mild), 2 (moderate), and 3 (severe). All continuous variables were tested for normality using the Shapiro-Wilk test. For normally distributed data, we used ANOVA and independent t-tests, while for non-normally distributed data, we employed the Kruskal-Wallis test and Mann-Whitney U test. Spearman correlation was used to assess associations between 25(OH)D and pulmonary function indices as well as other clinical indicators. Multivariable linear regression was used to evaluate independent associations after adjusting for confounders. Bonferroni correction was applied for multiple comparisons. All tests were two-sided, and P < 0.05 was considered statistically significant.

### 2.4 Random-forest model construction

Random-forest classification models were constructed as follows:

Data splitting: the dataset was randomly divided into a training set and a test set at a ratio of 7:3 (training set n = 63; test set n = 27).
Parameter setting: the number of trees (ntree) was set to 500; the number of variables randomly sampled at each split (mtry) was set to the square root of the number of variables; the splitting criterion was the Gini index.
Variable importance: importance was assessed using the mean decrease in Gini impurity.
Model optimization and evaluation: 5-fold cross-validation was performed on the training set; final performance was evaluated on the test set using accuracy, sensitivity, specificity, F1 score, and ROC curves; AUC and 95% CI were reported.

## 3 Results

### 3.1 Baseline characteristics

A total of 90 CKD patients were included. The mean age was 63.4 ± 12.3 years; 52 were male (57.8%) and 38 were female (42.2%). In total, 22 patients were in CKD stage 1, 12 in stage 2, 23 in stage 3, 15 in stage 4, and 18 in stage 5.

### 3.2 Correlation between serum 25(OH)D and pulmonary function indices

Correlation analysis (**Table 1**) showed that serum 25(OH)D concentrations were positively correlated with FEV1 (r = 0.257, P = 0.015) and FEV1% predicted (r = 0.463, P < 0.001), and were also positively correlated with small-airway indices FEF25 (r = 0.211, P = 0.046), FEF50 (r = 0.394, P < 0.001), and FEF75 (r = 0.354, P = 0.001). Serum 25(OH)D was positively correlated with DLCO% predicted (r = 0.611, P < 0.001) and negatively correlated with diffusion impairment grade (r = -0.585, P < 0.001), suggesting that higher 25(OH)D levels were associated with less severe diffusion impairment. No significant correlation was observed with FEV1/FVC (P > 0.05).

**Table 1:** Correlation analysis between serum 25(OH)D and pulmonary function indices in CKD patients.

Variable	Spearman *r*	*P* value
FEV1	0.257*	0.015
FEV1% predicted	0.463**	<0.001
FEV1/FVC	0.02	0.851
FEF25	0.211*	0.046
FEF50	0.394**	<0.001
FEF75	0.354**	0.001
DLCO% predicted	0.611**	<0.001
Diffusion impairment grade (DLCO grade)	-0.585**	<0.001

*P < 0.05; **P < 0.01.

### 3.3 Correlation between serum 25(OH)D and other clinical indicators

Correlation analysis (**Table 2**) showed that serum 25(OH)D levels were positively correlated with nutritional indicators such as TP (r = 0.415, P < 0.001) and ALB (r = 0.548, P < 0.001), and with iron-metabolism indicators including TIBC (r = 0.429, P < 0.001), Fe (r = 0.378, P < 0.001), and UIBC (r = 0.360, P = 0.001). Serum 25(OH)D levels were also positively correlated with renal function (eGFR; r = 0.461, P < 0.001), and negatively correlated with CKD stage (r = -0.482, P < 0.001), creatinine (Crea; r = -0.486, P < 0.001), ACR (r = -0.411, P < 0.001), and uric acid (UA; r = -0.329, P = 0.002), suggesting that patients with poorer renal function and heavier proteinuria tended to have lower 25(OH)D levels.

**Table 2:** Correlation analysis between serum 25(OH)D and other clinical indicators in CKD patients.

Variable	Spearman *r*	*P* value
CKD stage	-0.482**	<0.001
eGFR	0.461**	<0.001
Crea	-0.486**	<0.001
UA	-0.329**	0.002
PTH	-0.350**	0.001
BNP	-0.457**	<0.001
ACR	-0.580**	<0.001
Urine microalbumin	-0.541**	<0.001
ALB	0.548**	<0.001
TP	0.415**	<0.001
TNT	-0.532**	<0.001
CK-MB	-0.253*	0.020
MYO	-0.384**	<0.001
WBC	-0.177	0.095
NE%	-0.175	0.099
PLT	0.076	0.476
Hbg	0.491**	<0.001
HCT	0.522**	<0.001
β2-MG	-0.479**	<0.001
Ca	0.622**	<0.001
Mg	-0.170	0.109
P	-0.319**	0.002
Na	-0.106	0.320
K	-0.307**	0.003
Cl	-0.371**	<0.001
CRP	-0.253*	0.016
TIBC	0.429**	<0.001
Fe	0.378**	<0.001
UIBC	0.360**	0.001
Folate	-0.123	0.250
VitB12	0.074	0.491
Ferritin	-0.161	0.139

*P < 0.05; **P < 0.01.

To further explore factors independently associated with serum 25(OH)D levels, a multivariable linear regression model was built. After adjusting for confounders such as age, sex, BMI, and smoking history, DLCO% predicted (standardized Beta = 0.402, P < 0.001) and ALB (standardized Beta = 0.424, P < 0.001) remained independently positively associated with serum 25(OH)D, while UA (standardized Beta = -0.281, P < 0.001) remained independently negatively associated (**Table 3**).

**Table 3:** Multivariable linear regression analysis of serum 25(OH)D in CKD patients.

Variable	*B*	SE	Standardized *Beta*	*t*	*P* value
DLCO%	0.213**	0.042	0.402	5.051	<0.001
ALB	0.864**	0.161	0.424	5.382	<0.001
UA	-0.030**	0.008	-0.281	-3.654	<0.001
CRP	0.128*	0.061	0.159	2.100	0.039

*P < 0.05; **P < 0.01.

### 3.4 Multivariable regression analyses for pulmonary function indices

Multivariable linear regression models were constructed for each pulmonary function index to identify independent clinical factors associated with the outcomes.

Multivariable regression for FEV1 (**Table 4**) showed that height (Beta = 0.707, P < 0.001), ACR (Beta = -0.346, P < 0.001), age (Beta = -0.207, P = 0.001), smoking history (Beta = 0.177, P = 0.014), PLT (Beta = 0.178, P = 0.004), HCT (Beta = -0.226, P = 0.006), and β2-MG (Beta = -0.156, P = 0.013) were independently associated with FEV1.

**Table 4:** Multivariable linear regression analysis for FEV1.

Variable	*B*	SE	Standardized *Beta*	*t*	*P* value	95% *CI* (Lower)	95% *CI* (Upper)
Height	5.454**	0.573	0.707	9.521	<0.001	4.311	6.597
ACR	-0.002**	<0.001	-0.346	-4.793	<0.001	-0.003	-0.001
Age	-0.013**	0.004	-0.207	-3.533	0.001	-0.021	-0.006
Smoking history	0.007*	0.003	0.177	2.520	0.014	0.002	0.013
PLT	0.002**	0.001	0.178	2.994	0.004	0.001	0.004
HCT	-0.025**	0.009	-0.226	-2.854	0.006	-0.042	-0.008
β2-MG	-0.010*	0.004	-0.156	-2.550	0.013	-0.019	-0.002

*P < 0.05; **P < 0.01.

Multivariable regression for FEV1% predicted (**Table 5**) showed that Hgb (Beta = -1.515, P < 0.001), K (Beta = -0.421, P < 0.001), folate (Beta = -1.226, P < 0.001), MYO (Beta = 0.994, P = 0.003), and a history of hypertension (Beta = -0.218, P = 0.014) were independently associated with FEV1% predicted.

**Table 5:** Multivariable linear regression analysis for FEV1% predicted.

Variable	*B*	SE	Standardized *Beta*	*t*	*P* value	95% *CI* (Lower)	95% *CI* (Upper)
Hbg	-1.355**	0.283	-1.515	-4.791	<0.001	-1.918	-0.791
K	-14.636**	3.884	-0.421	-3.768	<0.001	-22.379	-6.894
Folate	-0.638**	0.169	-1.226	-3.769	<0.001	-0.976	-0.301
MYO	0.055**	0.018	0.994	3.048	0.003	0.019	0.091
Hypertension	-9.709*	3.868	-0.218	-2.510	0.014	-17.419	-1.999

*P < 0.05; **P < 0.01.

Multivariable regression for small-airway indices (FEF25/50/75) (**Tables 6**–**8**) showed that ACR (Beta = -0.356 and -0.452; P = 0.001 and < 0.001) and NE% (Beta = -0.308 and -0.219; P = 0.003 and 0.031) were independently negatively associated with FEF25 and FEF50, respectively. Serum 25(OH)D (Beta = 0.296, P = 0.007) was independently positively associated with FEF75, while hypertension (Beta = -0.252, P = 0.020) was independently negatively associated with FEF75.

**Table 6:** Multivariable linear regression analysis for FEF25.

Variable	*B*	SE	Standardized *Beta*	*t*	*P* value	95% *CI* (Lower)	95% *CI* (Upper)
ACR	-0.085**	0.024	-0.356	-3.535	0.001	-0.134	-0.037
NE%	-0.854**	0.279	-0.308	-3.056	0.003	-1.410	-0.297

**P < 0.01.

**Table 7:** Multivariable linear regression analysis for FEF50.

Variable	*B*	SE	Standardized *Beta*	*t*	*P* value	95% *CI* (Lower)	95% *CI* (Upper)
ACR	-0.105**	0.023	-0.452	-4.559	<0.001	-0.150	-0.059
NE%	-0.585*	0.265	-0.219	-2.204	0.031	-1.113	-0.056

*P < 0.05; **P < 0.01.

**Table 8:** Multivariable linear regression analysis for FEF75.

Variable	*B*	SE	Standardized *Beta*	*t*	*P* value	95% *CI* (Lower)	95% *CI* (Upper)
25(OH)D	0.601**	0.215	0.296	2.796	0.007	0.173	1.029
Hypertension	-13.917*	5.858	-0.252	-2.376	0.020	-25.585	-2.249

*P < 0.05; **P < 0.01.

Multivariable regression for DLCO% predicted (**Table 9**) showed that serum 25(OH)D (Beta = 0.391, P < 0.001) was independently positively associated with DLCO% predicted, while CKD stage (Beta = -0.567, P < 0.001) was independently negatively associated. Smoking history (Beta = 0.195, P = 0.016) showed a positive association with DLCO% predicted, but the study did not further distinguish cumulative smoking exposure or secondhand smoke exposure.

**Table 9:** Multivariable linear regression analysis for DLCO% predicted.

Variable	*B*	SE	Standardized *Beta*	*t*	*P* value	95% *CI* (Lower)	95% *CI* (Upper)
25(OH)D	0.738**	0.147	0.391	5.016	<0.001	0.445	1.031
CKD stage	-6.405**	1.146	-0.567	-5.591	<0.001	-8.689	-4.120
Weight	0.324*	0.123	0.202	2.634	0.010	0.079	0.569
PLT	0.152**	0.032	0.338	4.725	<0.001	0.088	0.217
WBC	-3.310**	1.014	-0.228	-3.264	0.002	-5.333	-1.288
CO2 combining power (CO2CP)	-2.240**	0.641	-0.341	-3.492	0.001	-3.519	-0.961
Smoking history	0.256*	0.104	0.195	2.476	0.016	0.050	0.463

*P < 0.05; **P < 0.01.

Multivariable regression for diffusion impairment grade (**Table 10**) suggested that higher serum 25(OH)D levels (Beta = -0.543, P < 0.001) were independently associated with a lower diffusion impairment grade.

**Table 10:** Multivariable linear regression analysis for diffusion impairment grade.

Variable	*B*	SE	Standardized *Beta*	*t*	*P* value	95% *CI* (Lower)	95% *CI* (Upper)
25(OH)D	-0.047**	0.007	-0.543	-7.104	<0.001	-0.060	-0.034
VitB12	0.001**	<0.001	0.218	3.300	0.002	<0.001	0.002
Weight	-0.022**	0.006	-0.301	-3.604	0.001	-0.034	-0.010
PLT	-0.007**	0.001	-0.321	-4.975	<0.001	-0.009	-0.004
Mg	1.492**	0.534	0.186	2.793	0.007	0.426	2.557
ACR	0.002**	0.001	0.231	3.224	0.002	0.001	0.003
TIBC	0.003**	0.001	0.188	2.869	0.005	0.001	0.006
Smoking history	-0.016**	0.005	-0.272	-3.228	0.002	-0.027	-0.006
Sex	-0.389*	0.189	-0.201	-2.057	0.043	-0.767	-0.012

*P < 0.05; **P < 0.01.

### 3.5 Random-forest models for predicting pulmonary function decline in CKD

**Figure 1:**
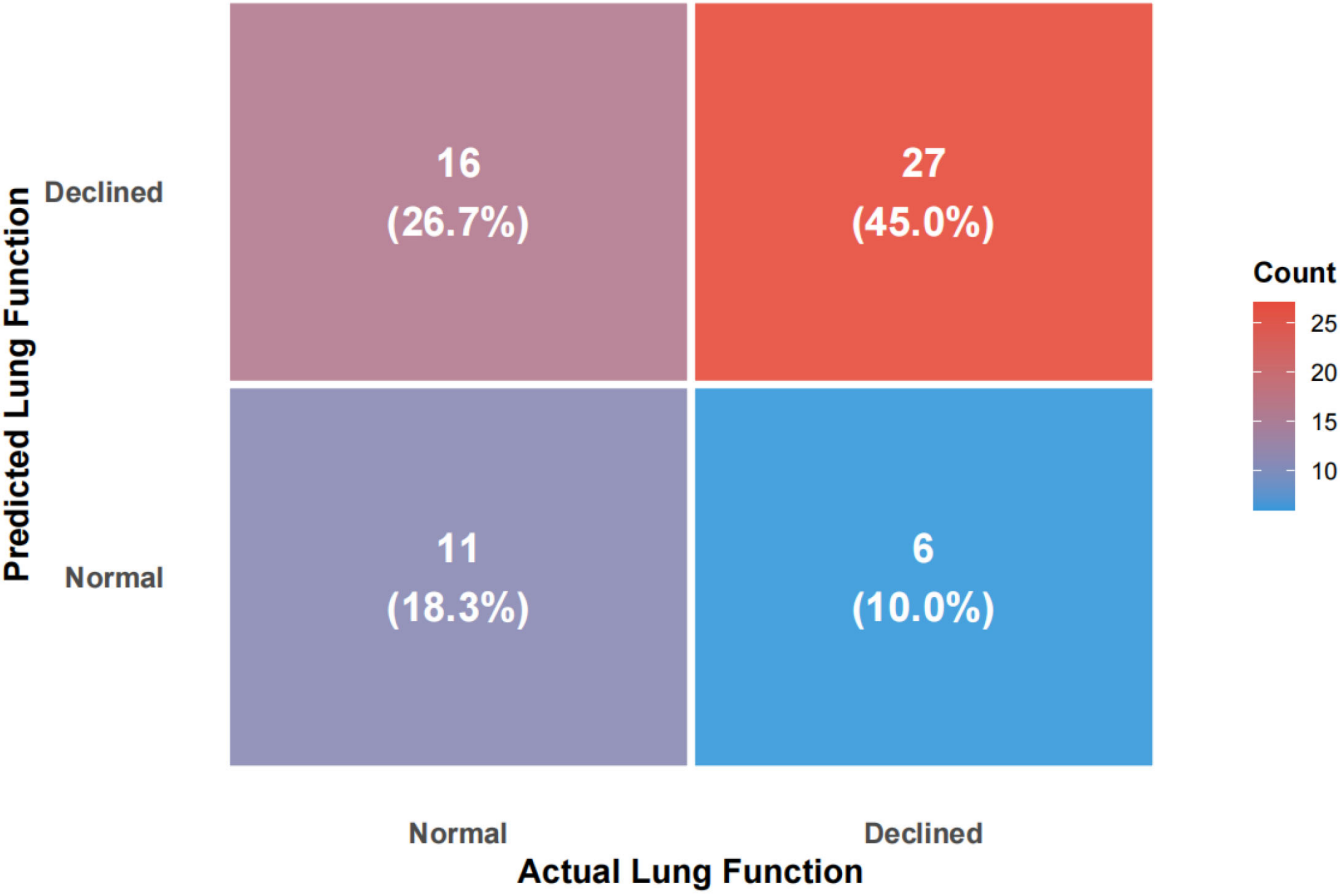
Confusion matrix of the random forest model on the test set.

**Figure 2:**
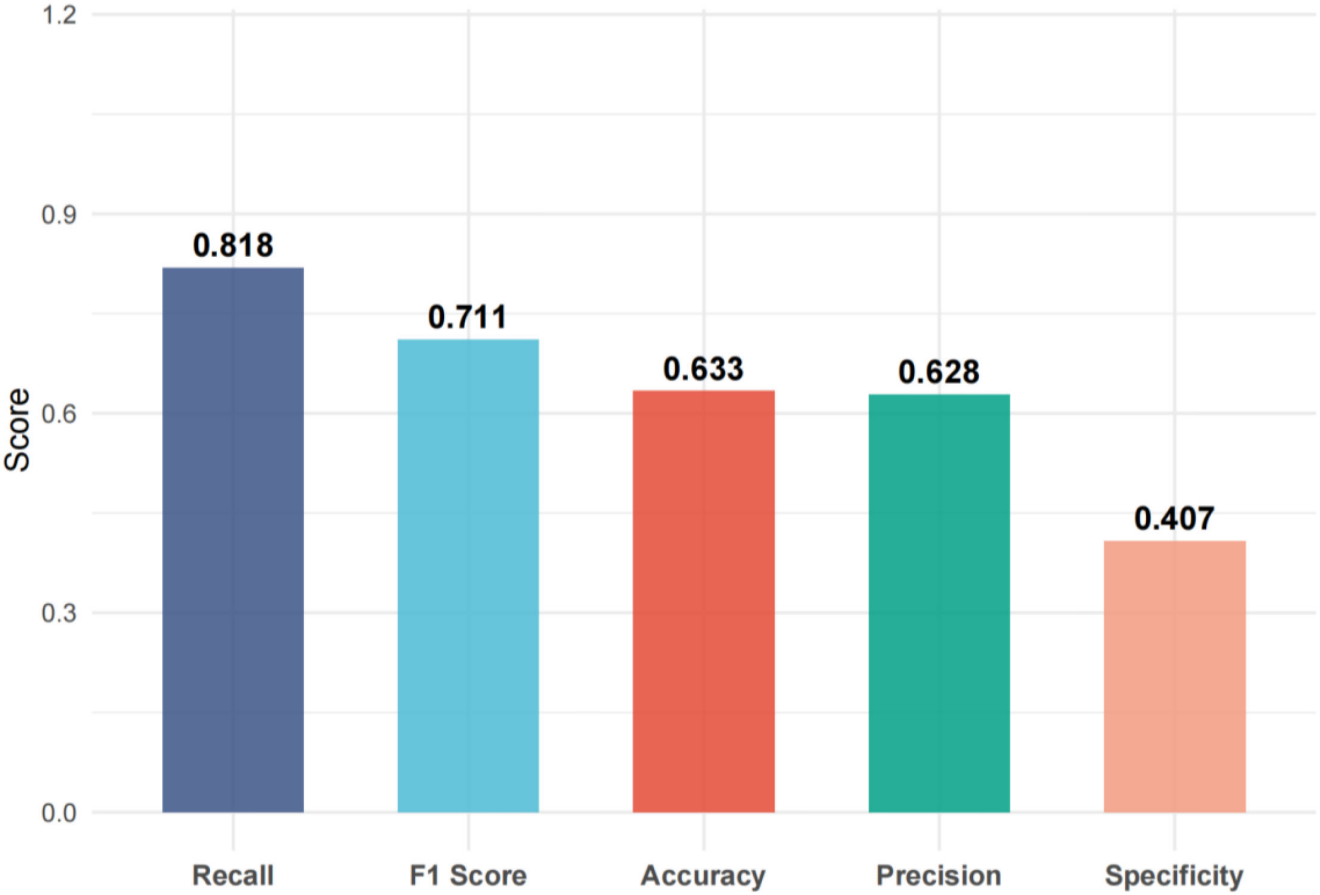
Model performance metrics.

**Figure 3:**
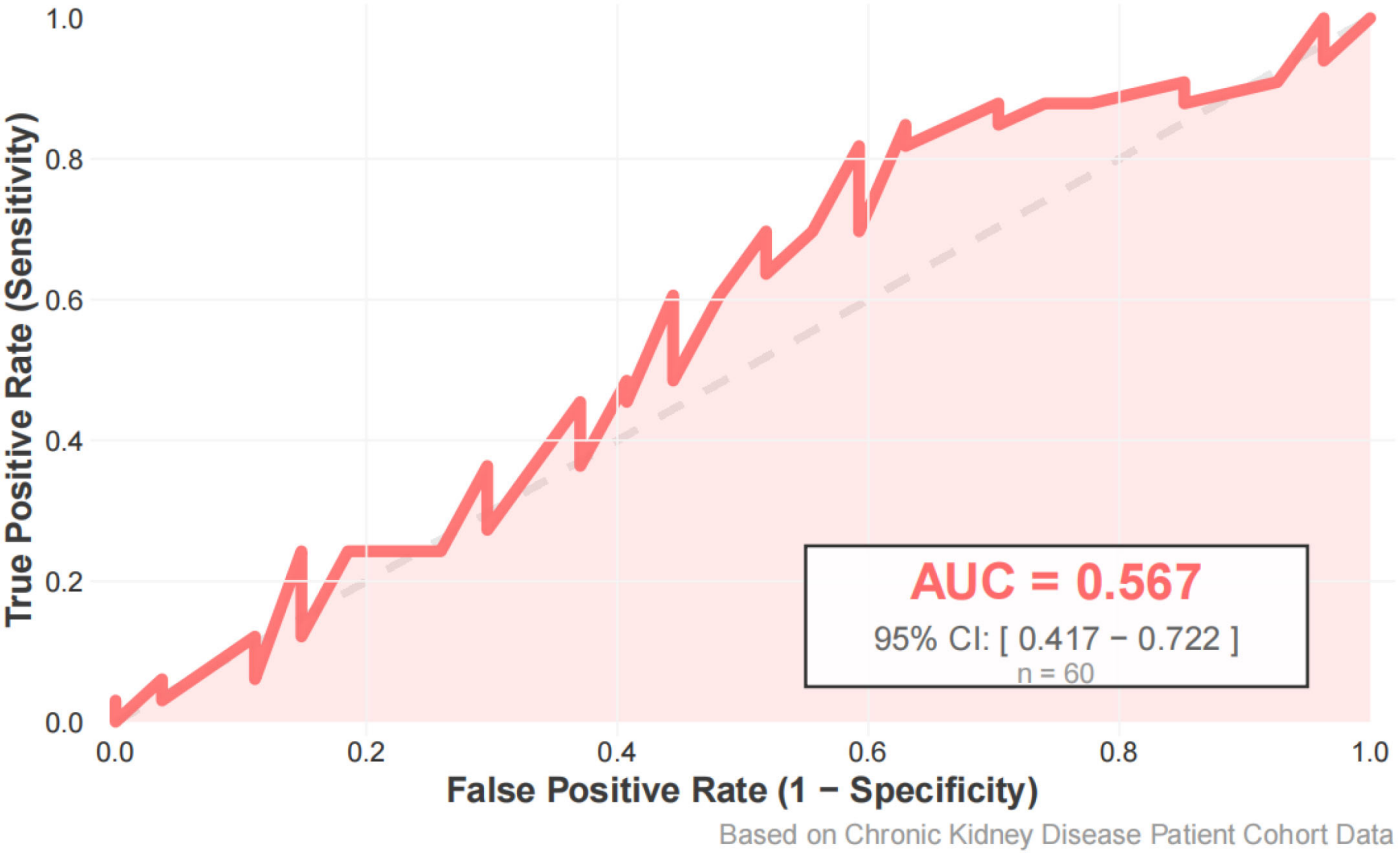
ROC curve of the random forest model distinguishing lung function decline in the test set.

**Figure 4:**
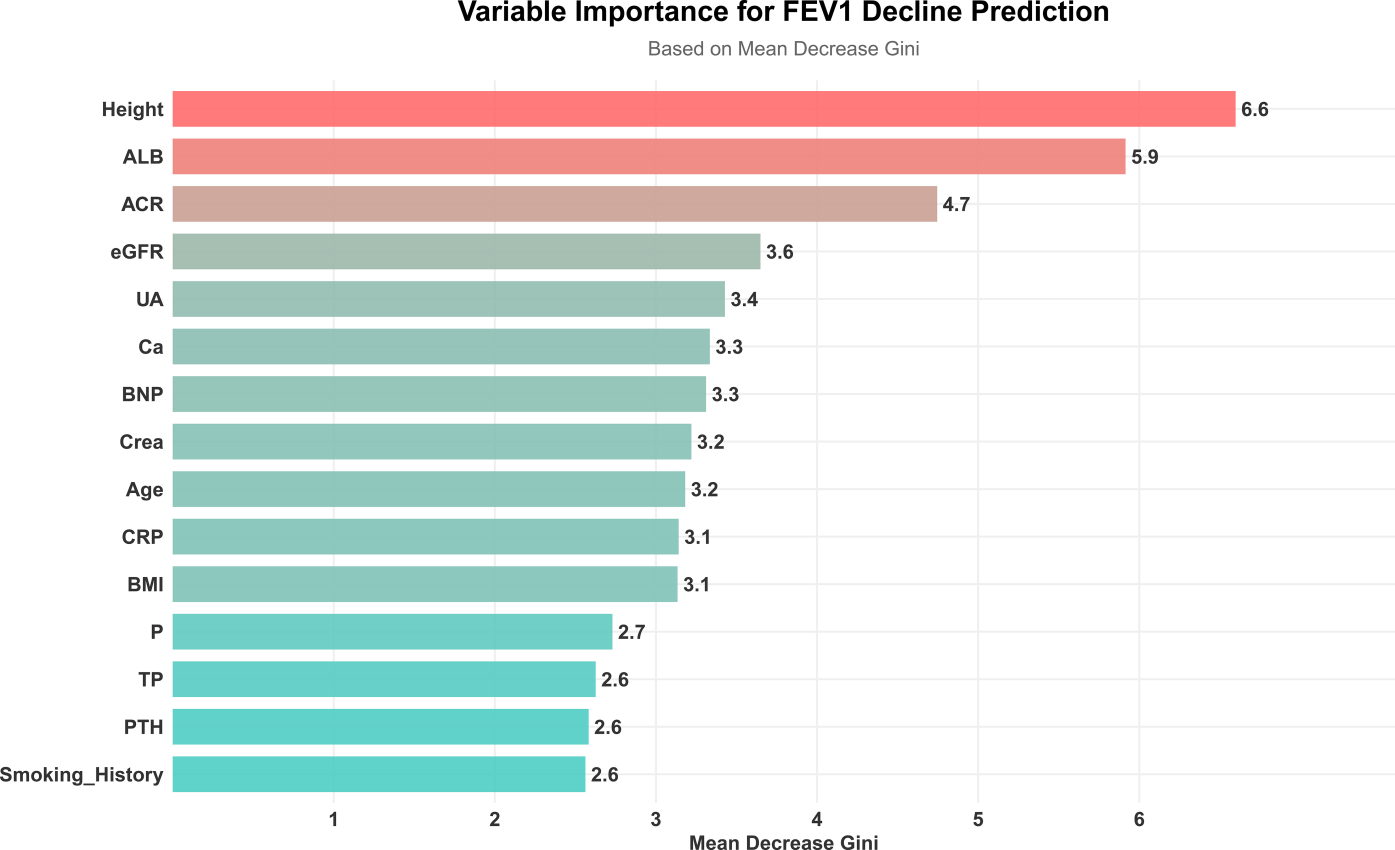
Parameter importance ranking in random forest model.

Using FEV1% predicted as the criterion for ventilatory impairment (FEV1% predicted < 80% defined as “low lung function/ventilatory impairment”), the outcome was dichotomized and a random-forest model was constructed. The model achieved an accuracy of 63.3%, sensitivity of 81.8%, specificity of 40.7%, and F1 score of 0.711 on the test set (**Figures 1**, **2**). ROC analysis (**Figure 3**) yielded an AUC of 0.567 (95% CI: 0.417-0.722). Variable-importance ranking (**Figure 4**) showed that kidney-function-related indicators such as ACR, eGFR, UA, and serum creatinine were among the top contributors.

**Figure 5:**
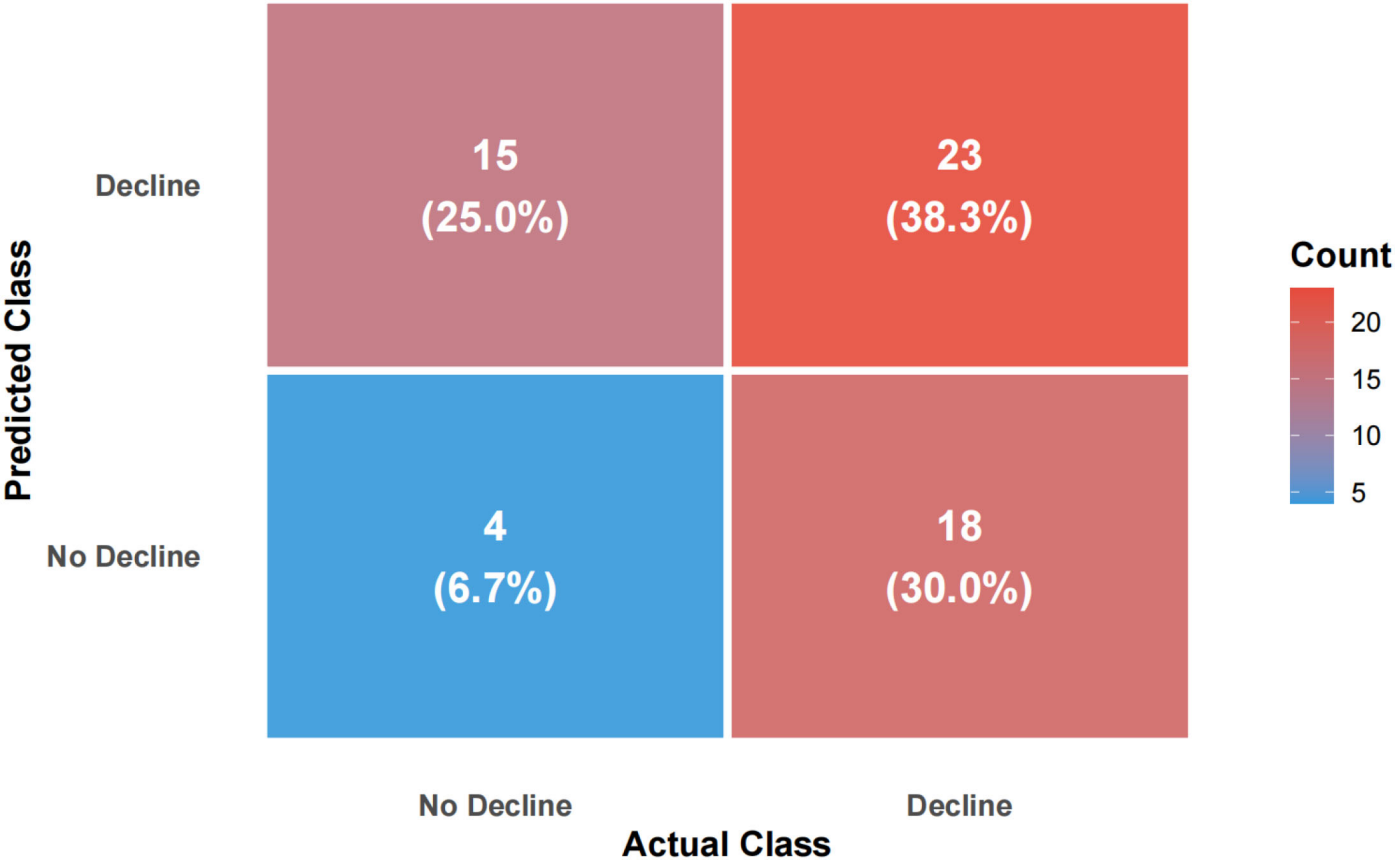
Confusion matrix of the random forest model on the test set.

**Figure 6:**
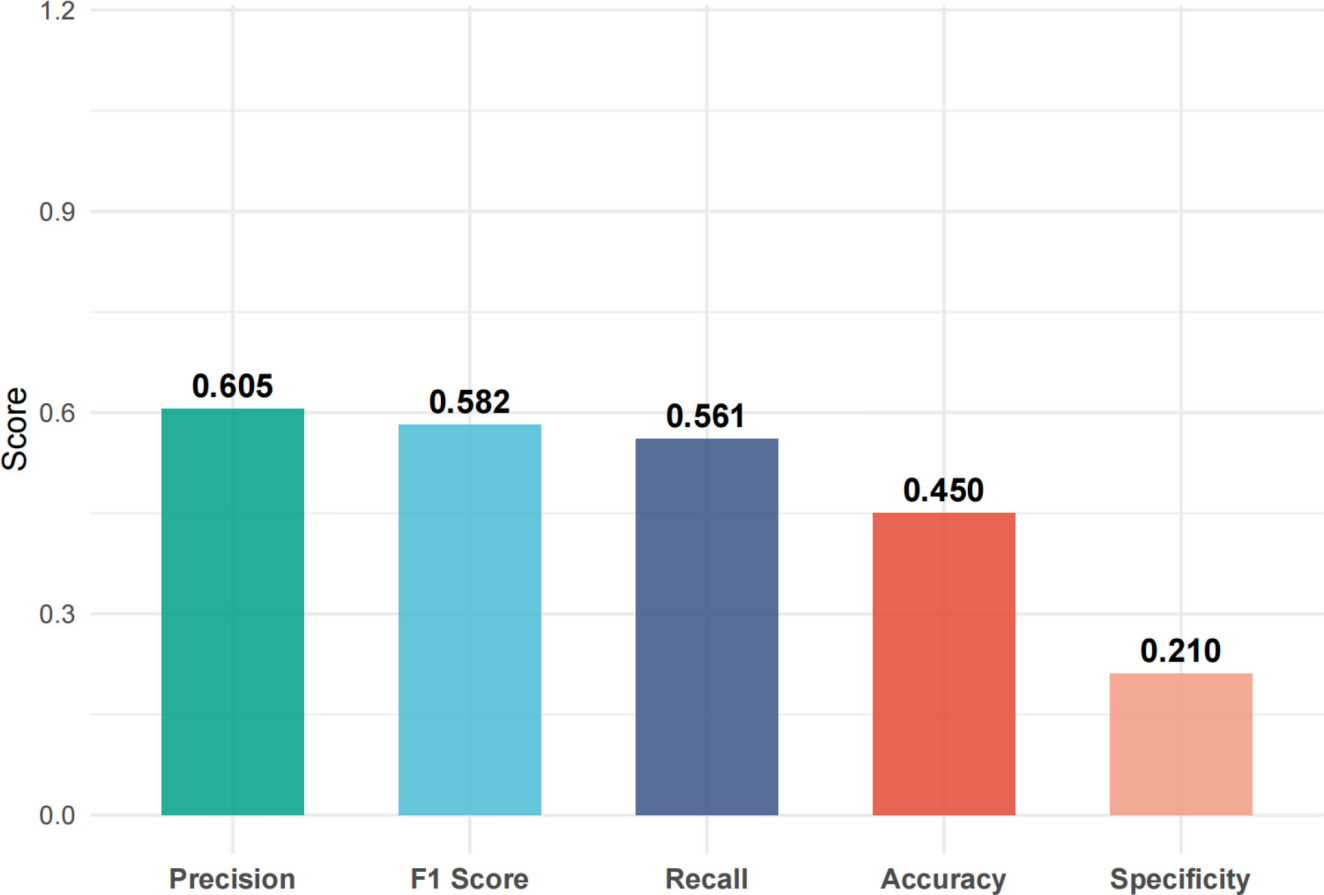
Model performance metrics.

**Figure 7:**
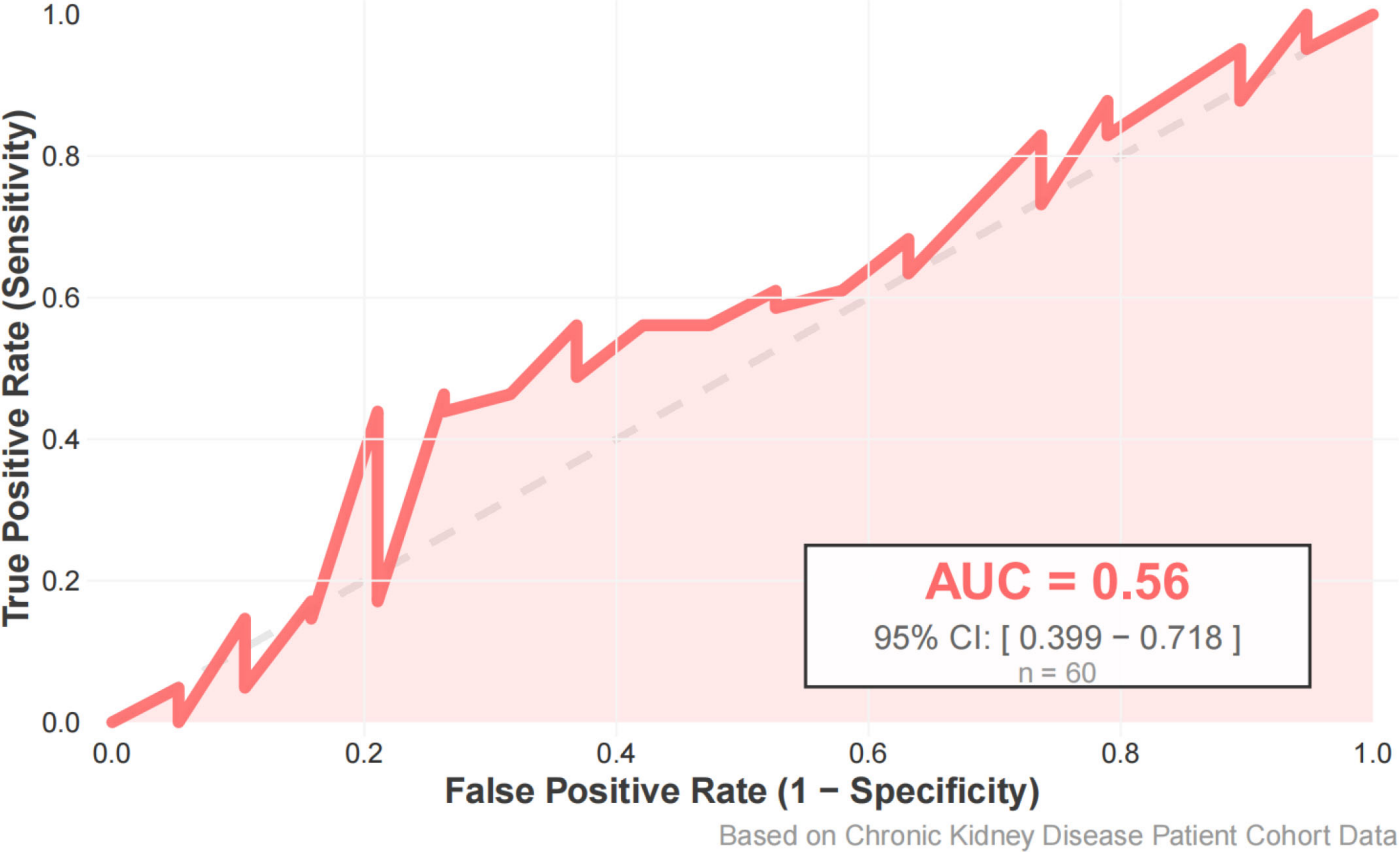
ROC curve of the random forest model distinguishing lung function decline in the test set.

**Figure 8:**
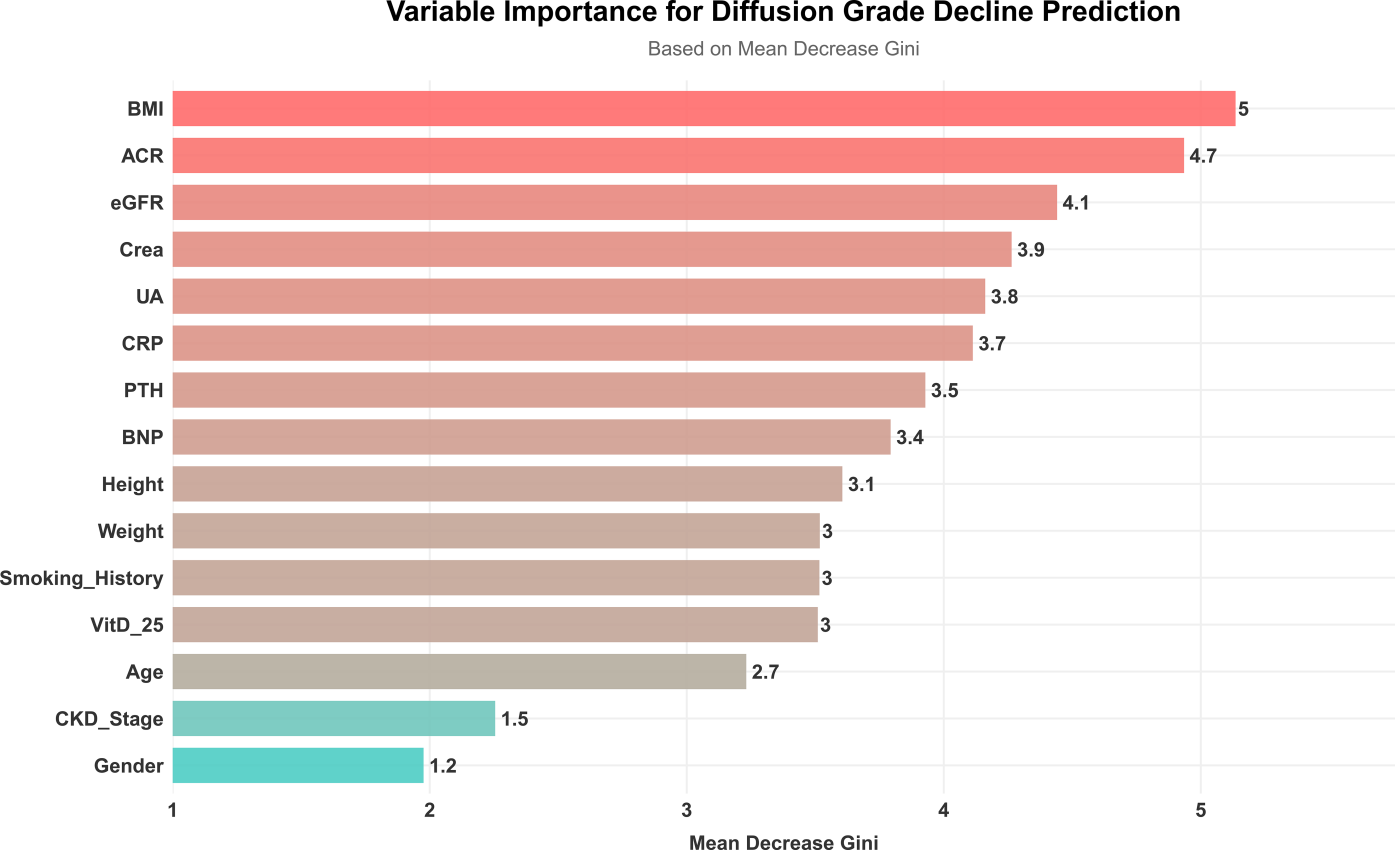
Parameter importance ranking in random forest model.

Using diffusion impairment grade (normal as the reference group; mild, moderate, and severe grouped as the impaired group) as the prediction target, another random-forest model was constructed. The model achieved an accuracy of 60.5%, sensitivity of 56.1%, specificity of 21.0%, and F1 score of 0.582 on the test set (**Figures 5**, **6**). ROC analysis (**Figure 7**) showed an AUC of 0.560 (95% CI: 0.399-0.718). In the variable-importance ranking (**Figure 8**), ACR, eGFR, serum creatinine, and UA also ranked highly.

Both models had AUC values close to 0.56 with limited overall discrimination, indicating that the current combination of variables was not ideal for predicting pulmonary function decline in CKD patients. Potential reasons include the relatively small sample size and the complex etiology of pulmonary function decline in CKD involving uremic toxins, inflammation, malnutrition, cardiovascular complications, and multi-system interactions; clinical biochemical indicators alone may not fully capture the core determinants.

## 4 Discussion

In this cross-sectional study, we observed associations between serum 25(OH)D levels and multiple pulmonary function indices in hospitalized CKD patients, particularly FEV1% predicted and diffusion function. This finding is consistent with multiple biological effects of 25(OH)D in the lung, including anti-inflammatory, antioxidative, anti-fibrotic effects and maintenance of endothelial function (11, 12) 25(OH)D may alleviate chronic pulmonary inflammation by down-regulating the NF-κB pathway (13), and may improve gas-exchange function by regulating transforming growth factor-β (TGF-β) signaling, thereby inhibiting alveolar-septal fibrosis and capillary endothelial injury (14). This study further confirmed that even after adjusting for confounders such as age, sex, BMI, and Hgb, 25(OH)D remained an independent influencing factor for DLCO% and FEF75 (15) suggesting a potential independent role in maintaining pulmonary function in CKD (16). It should be noted that evidence suggesting vitamin D supplementation may improve diffusion function mainly comes from other populations, and whether this can be extrapolated to CKD populations requires dedicated validation.

Beside 25(OH)D, this study identified other key factors influencing pulmonary function in CKD. As a quantitative indicator of proteinuria, ACR was an independent negative factor in multiple regression models for FEV1, FEF25, FEF50, DLCO% predicted, and diffusion impairment grade. This suggests that protein malnutrition and/or tubulointerstitial injury accompanied by systemic micro-inflammation may broadly impair ventilatory and diffusion functions by affecting respiratory muscle strength, pulmonary tissue repair capacity, and exacerbating oxidative stress (17, 18) In addition, associations between core renal indicators (e.g., eGFR and Crea) and pulmonary function, as well as their high importance ranking in the predictive models, support the existence of a “lung-kidney axis” (19). Declining renal function leads to accumulation of uremic toxins, acid-base and electrolyte disturbances, increased volume load, and anemia, forming the pathological basis of CKD-related pulmonary dysfunction (20, 21). Our results support the view that 25(OH)D may act as a “regulator” or “mediator” along this axis (21).Its deficiency is both a consequence of renal impairment and may also exacerbate systemic pathological changes (e.g., inflammation and malnutrition), thereby amplifying pulmonary injury (22, 23).

This study also explored random-forest models for predicting pulmonary function decline in CKD. Although the models demonstrated certain significance (especially higher sensitivity of the FEV1% model), overall specificity was limited. This reflects the complexity of pulmonary function decline in CKD. Future work should: (1) expand sample size and conduct multi-center validation to improve robustness and generalizability; (2) incorporate additional variables closer to respiratory physiology and exposure history (e.g., detailed smoking exposure, lung imaging, dialysis-related factors, and medication use) to better capture the full disease picture; and (3) explore ensembles of multiple machine-learning algorithms or deep-learning approaches to parse higher-order interactions among variables.

Limitations: (1) The cross-sectional design does not allow for causal inference between 25(OH)D and pulmonary function. (2) All samples were collected from a single center and consisted of hospitalized patients, which may introduce selection bias. (3) Certain important confounders were not fully included, such as vitamin D supplementation, sunlight exposure, dialysis status, and more detailed smoking exposure. (4) Although the sample size met basic requirements for statistical analyses, it remained insufficient for constructing high-performance predictive models. Multi-center prospective studies are needed for further validation.

## 5 Conclusions

This cross-sectional study systematically evaluated associations between serum 25(OH)D levels and multidimensional pulmonary function indices in hospitalized CKD patients. The results suggest that serum 25(OH)D levels are independently associated with multiple pulmonary function indices, especially diffusion-related indices. Variable importance ranking from the random forest model indicated significant contributions from renal function and proteinuria-related indicators, with 25(OH)D also showed some importance. However, given the limited sample size and overall model performance, the predictive results require validation in larger samples and external cohorts.

From a clinical perspective, this study suggests that assessment of 25(OH)D status and pulmonary function may have significance in certain high-risk CKD patients (e.g., those with malnutrition, anemia, marked proteinuria, and respiratory symptoms). Its benefit evaluation and whether routine screening is suggested requires higher-quality evidence.

Future research directions include: large-scale prospective cohort studies to clarify the causal relationship between 25(OH)D and pulmonary function impairment; randomized controlled trials to verify the efficacy and safety of 25(OH)D supplementation in improving pulmonary outcomes in CKD; and multi-omics approaches to explore molecular mechanisms within the “lung-kidney axis,” providing precise strategies to protect pulmonary function.

## Data Availability

The original contributions presented in the study are included in the article/Supplementary Material. Further inquiries can be directed to the corresponding author.
